# Voluntary Exercise Can Ameliorate Insulin Resistance by Reducing iNOS-Mediated S-Nitrosylation of Akt in the Liver in Obese Rats

**DOI:** 10.1371/journal.pone.0132029

**Published:** 2015-07-14

**Authors:** Takamasa Tsuzuki, Shohei Shinozaki, Hideko Nakamoto, Masao Kaneki, Sataro Goto, Kentaro Shimokado, Hiroyuki Kobayashi, Hisashi Naito

**Affiliations:** 1 Graduate School of Health and Sports Science, Juntendo University, Chiba, Japan; 2 Research Fellow of Japan Society for promotion of Science, Tokyo, Japan; 3 Department of Arteriosclerosis and Vascular Biology, Tokyo Medical and Dental University Graduate School of Medicine, Tokyo, Japan; 4 Institute of Health and Sports Science & Medicine, Juntendo University Graduate School of Health and Sports Science, Chiba, Japan; 5 Department of Anesthesia, Critical Care and Pain Medicine, Massachusetts General Hospital, Shriners Hospitals for Children, Harvard Medical School, Charlestown, Massachusetts, United States of America; 6 Department of Geriatrics and Vascular Medicine, Tokyo Medical and Dental University Graduate School of Medicine, Tokyo, Japan; 7 Department of General Medicine, Mito Medical Center, Tsukuba University Hospital, Ibaraki, Japan; Monash University, AUSTRALIA

## Abstract

Voluntary exercise can ameliorate insulin resistance. The underlying mechanism, however, remains to be elucidated. We previously demonstrated that inducible nitric oxide synthase (iNOS) in the liver plays an important role in hepatic insulin resistance in the setting of obesity. In this study, we tried to verify our hypothesis that voluntary exercise improves insulin resistance by reducing the expression of iNOS and subsequent S-nitrosylation of key molecules of glucose metabolism in the liver. Twenty-one Otsuka Long-Evans Tokushima Fatty (OLETF) rats, a model of type 2 diabetes mellitus, and 18 non-diabetic control Long-Evans Tokushima Otsuka (LETO) rats were randomly assigned to a sedentary group or exercise group subjected to voluntary wheel running for 20 weeks. The voluntary exercise significantly reduced the fasting blood glucose and HOMA-IR in the OLETF rats. In addition, the exercise decreased the amount of iNOS mRNA in the liver in the OLETF rats. Moreover, exercise reduced the levels of S-nitrosylated Akt in the liver, which were increased in the OLETF rats, to those observed in the LETO rats. These findings support our hypothesis that voluntary exercise improves insulin resistance, at least partly, by suppressing the iNOS expression and subsequent S-nitrosylation of Akt, a key molecule of the signal transduction pathways in glucose metabolism in the liver.

## Introduction

With the excess consumption of food and physical inactivity as well as advancing population aging, the incidence of lifestyle-related diseases, including metabolic syndrome, is rapidly and broadly increasing. However, studies to prevent these diseases have only recently been initiated in human. Caloric restriction and exercise are commonly recommended for the prevention and amelioration of obesity and lifestyle-related diseases [1]. It is important to note, however, that caloric restriction and resultant prevention of obesity does not always successfully reverse insulin resistance [2].

Chronic inflammation is a common etiology in patients with lifestyle-related diseases, such as diabetes or cardiovascular disease, or the aging process itself [3]. A new post-translational protein modification mechanism, termed S-nitrosylation, has recently been identified, in which the nitric oxide (NO) produced by conditions of chronic inflammation is covalently attached to the cysteine residue [4]. In addition, protein S-nitrosylation has recently been reported to be a regulatory component of signal transduction comparable to phosphorylation [5].

We and others have shown that inducible nitric oxide synthase (iNOS) inhibitor treatment improves insulin signaling at the level of insulin receptor substrate-1 (IRS-1) and -2 and Akt in the liver in genetically obese diabetic (ob/ob) mice [6, 7]. iNOS and NO donors reversibly inactivate Akt via S-nitrosylation *in vitro* and in intact cells without altering the phosphorylation status at threonine 308 or serine 473 [8]. In addition, we previously reported that the overexpression of iNOS contributes to hepatic insulin resistance via the S-nitrosylation of insulin signaling molecules [9]. The reverse process of S-nitrosylation is called denitrosylation, which can occur spontaneously in the presence of the reduced form of glutathione (GSH) [10]. Moreover, we previously showed that regular exercise increases the intracellular GSH content and decreases chronic low-level inflammation in the liver in aged rats [11]. It has also been reported that treatment with a small-molecule activator of the transcription factor nuclear factor erythroid 2-related factor 2 (NRF2) denitrosylates HDAC2 by upregulating the GSH level [12]. Therefore, denitrosylation is facilitated when the intracellular GSH content is increased.

Otsuka Long-Evans Tokushima Fatty (OLETF) rats constitute a model of obesity and type 2 diabetes and are selectively bred for the null expression of the cholecystokinin-1 receptor [13, 14]. Sedentary OLETF rats show insulin resistance at 10–20 weeks of age and type 2 diabetes at approximately 30 weeks of age [13, 15]. However, OLETF rats subjected to voluntary exercise display suppressed body weight gain [13] and improved insulin sensitivity [16]. OLETF and Long-Evans Tokushima Otsuka (LETO) rats are well studied with respect to obesity-induced insulin resistance and the beneficial effects of regular physical activity [13, 14, 17].

Numerous studies have shown that regular exercise prevents obesity and insulin resistance, whereas sedentary behavior increases the risk of metabolic syndrome [18–22]. The potential mechanisms by which exercise prevents obesity-induced insulin resistance include the transport glucose of into skeletal muscles [23, 24]. While increased glucose transportation is maintained for at least five days after exercise [24]. Therefore, other factors (e.g., hepatic and adipose tissue insulin resistance) atop of muscle insulin resistance may participate in the improvements in “whole-body” insulin sensitivity induced by regular exercise. Of note, hepatic insulin resistance plays a crucial role in hyperglycemia. Whereas muscle-specific insulin receptor knockout does not induce hyperglycemia or hyperinsulinemia [25], hepatocyte-specific insulin receptor knockout mice exhibit overt hyperglycemia and hyprerinsulinemia [26]. The reversal of hyperglycemia and hyperinsulinemia cannot be accounted for by the improvement in muscle insulin resistance alone. Hence, in this study, we assessed the mechanisms by which regular exercise improves insulin resistance in the liver.

We hypothesized that voluntary exercise improves insulin resistance by reducing the expression of iNOS and subsequent S-nitrosylation of key molecules of glucose metabolism in the liver. In order to evaluate this hypothesis, we tested whether voluntary exercise prevents hepatic insulin resistance in OLETF and LETO rats.

## Materials and Methods

### Materials

Methyl methanethiosulfonate (MMTS), ascorbate sodium, dithiothreitol (DTT), glutathione reduced ethyl ester (GSH-MEE) (Sigma, St. Louis, MO), N-(6-(biotinamido)hexyl)-3'-(2'-pyridyldithio)-propionamide (HPDP-biotin), NeutrAvidin agarose resins (Pierce, Rockford, IL), anti-Akt, anti-phospho-Akt (T308), anti-phospho-Akt (S473), anti-SAPK/JNK, anti-phospho-SAPK/JNK antibodies (Cell Signaling, Beverly, MA), anti-IRS-1 and anti-phospho-IRS-1 (S307) antibodies (Santa Cruz Biotechnology, Santa Cruz, CA) were purchased commercially.

### Animals

The study protocol was approved by the Juntendo University Animal Care Committee and was conducted according to the guiding principles for the Care and Use of Laboratory Animals set forth by the Physiological Society of Japan. Four-week-old Otsuka Long-Evans Tokushima Fatty (OLETF) and Long-Evans Tokushima Otsuka (LETO) rats were purchased from Japan SLC (Shizuoka, Japan). At five weeks of age, both the OLETF and LETO rats were randomly assigned to a sedentary group (OLETF-SED, LETO-SED) or voluntary exercise group (OLETF-VE, LETO-VE) for 20 weeks. The rats were housed in an environment-controlled animal facility (24 ± 1°C, 55 ± 5%) and illuminated with a 12:12-hour light-dark cycle. The animals were provided standard rodent chow and water ad libitum. The rats in the voluntary exercise group were granted free access to a running wheel during the experimental period.

### Glucose tolerance test

Glucose (1.0 g/kg BW) was intraperitoneally administered to the LETO and OLETF rats following overnight fasting at 25 weeks of age. Blood samples were collected just before and at 30, 60 and 120 minutes after glucose injection. The blood glucose levels were measured using the Glutest Neo Super device (Sanwa Kagaku Kenkyusho, Aichi, Japan). The plasma insulin concentrations were determined using an AKRIN-010S rat insulin ELISA kit (Shibayagi, Gunma, Japan).

### Assessment of insulin sensitivity according to the homeostasis model assessment (HOMA) and insulin resistance (IR) index

In order to assess the whole-body insulin sensitivity in the LETO and OLETF rats, the HOMA-IR index was determined using the HOMA2 Calculator software program (downloaded from www.OCDEM.ox.ac.uk) based on the blood glucose and plasma insulin concentrations at 25 weeks of age.

### Biotin-switch assay to detect the S-nitrosylation of insulin signaling molecules

The degree of S-nitrosylation of Akt was evaluated according to a biotin-switch assay, as previously described [9] with minor modifications. Briefly, the liver tissues were rinsed with phosphate-buffered saline (PBS), powdered under liquid nitrogen and homogenized in homogenization buffer (PBS-HCl, pH 3.5, 150 mM NaCl, 1 mM EDTA, 1 mM diethylenetriaminepentaacetic acid [DTPA], 2.5% SDS, 0.5% NP-40, 0.1 mM neocuproine, 80 μM of carmustine, 1 mM PMSF, protease inhibitor cocktail [Sigma]). The homogenates were subsequently incubated at 50°C for 20 minutes with vortexing every two minutes following the addition of 1 volume of blocking buffer (PBS-HCl, pH 3.5, 150 mM NaCl, 1 mM EDTA, 1 mM DTPA, 2.5% SDS, 0.1 mM neocuproine, 40 mM MMTS). The proteins were precipitated with pre-chilled acetone, dissolved in modified HENS buffer (25 mM HEPES, pH 7.7, 1% SDS, 1 mM EDTA, 1 mM DTPA, 0.1 mM neocuproine) and neutralized with HEN buffer (25 mM HEPES, pH 7.7, 0.5% Triton X-100, 1 mM EDTA, 1 mM DTPA, 100 mM NaCl, 0.1 mM neocuproine). The samples were then incubated with 4 mM HPDP-biotin in the presence of 4 mM ascorbate sodium for one hour at room temperature. After excess HPDP-biotin was removed via acetone precipitation, the samples were incubated with streptavidin-agarose beads for one hour at room temperature. The beads were then washed three times with wash buffer (25 mM HEPES, pH 7.7, 1 mM EDTA, 500 mM NaCl, 0.5% Nonidet P-40), and the biotinylated proteins were eluted via incubation with elution buffer (20 mM HEPES, pH 7.7, 1 mM EDTA, 100 mM NaCl, 200 mM DTT) for 30 minutes and separated via SDS-PAGE for immunoblotting with the anti-Akt or anti-IRS-1 antibody.

### Evaluation of the effects of voluntary exercise on hepatic insulin signaling

To assess the hepatic insulin sensitivity in OLETF rats, we injected insulin via the portal vein in both sedentary and voluntary exercise groups. At five weeks of age, the OLETF rats were randomly assigned to a sedentary or voluntary exercise group. After 10-week voluntary exercise or lack thereof, the rats were fasted overnight at 15 weeks of age. Under anesthesia, insulin (0.5 units/Kg BW, Humalin R; Eli lilly, Indiana, IN) or saline was injected via the portal vein. Five minutes after the injection, the liver was removed and snap frozen in liquid nitrogen.

### Immunoblotting

The liver samples were homogenized as previously described [9], with minor modifications. Briefly, the tissues were homogenized in ice-cold homogenization buffer A (50 mM HEPES, pH 8.0, 150 mM NaCl, 2 mM EDTA, 2.5% lithium dodecylsulfate, 2% CHAPS, 10% glycerol, 10 mM sodium fluoride, 2 mM sodium vanadate, 1 mM PMSF, 10 mM sodium pyrophosphate, 1 mM DTT, protease inhibitor cocktail). Following incubation at 4°C for 30 minutes, the homogenized samples were centrifuged at 13,000 g for 10 minutes at 4°C. Immunoblotting was subsequently performed as previously described [27]. ECL select reagent (GE Healthcare) was then used to visualize the blots, and bands of interest were scanned using the LAS 1000 (Fujifilm, Carson, Japan) and quantified according to the NIH Image 1.62 software program (NTIS, Springfield, VA).

### Total RNA isolation and quantitative RT-PCR

Total RNA was isolated using an RNeasy Mini kit (Qiagen, Valencia, CA), and first-strand cDNA was synthesized from 1 μg of total RNA using a High Capacity cDNA Reverse Transcription Kit (Applied Biosystems, Carlsbad, CA). The real-time RT-PCR analyses were performed as previously described [28] with 10 ng of cDNA and TaqMan probes (Applied Biosystems) for inducible nitric oxide synthase (*iNos* as knoen as *Nos2*), sterol regulatory element binding transcription factor 1 (*Srebp1*), stearoyl-Coenzyme A desaturase 1(*Scd1*), acetyl-CoA carboxylase alpha (*Acc*), fatty acid synthase (*Fas*), glycerol-3-phosphate acyltransferase 2 (*Gpat2*) and 18S ribosomal RNA using a Thermal Cycler Dice (Takara, Osaka, Japan). The gene expression of iNOS was normalized to that of 18S ribosomal RNA.

### Measurement of the GSH/GSSG ratio

The reduced glutathione (GSH) to oxidized glutathione (GSSG) ratio was measured using the Bioxytech GSH/GSSG Assay kit (Percipio Biosciences, Foster, CA) according to the manufacturer’s instructions. Briefly, the liver tissues were homogenized in 15-fold volumes of ice-cold 5% metaphosphoric acid (MPA), and the homogenates were centrifuged at 10,000 g for 20 minutes at 4°C. For the GSH analysis, MPA extracts were diluted 60-fold in assay buffer (Na•PO_4_ with EDTA). The final concentration in the samples was 1/488. For the GSSG analysis, M2VP was mixed with the MPA extracts to inhibit the oxidation of GSH to GSSG, and the samples were neutralized by the addition of 5 μl of TEA and subsequently diluted 4-fold in 5% MPA and 15-fold in assay buffer. The final concentration in the samples was 1/60. The changes in absorbance at 412 nm were recorded for three minutes, and the GSH/GSSG ratio was calculated based on the formula described in the manufacturer’s instructions.

### Measurement of the liver TG content

Lipid extraction from the liver tissues was performed as previously described [29]. The triglyceride content in the lipid extracts was measured using the Triglyceride E-test Wako kit (WAKO) according to the manufacturer’s instructions.

### Measurement of the plasma leptin concentrations

The plasma leptin concentrations were measured using the AKRIN-010S rat insulin ELISA kit (Shibayagi, Gunma, Japan) according to the manufacturer’s instructions.

### Statistical analysis

The data were compared using unpaired *t* test or one-way or two-way analysis of variance followed by Scheffe’s multiple comparison test. A P value of <0.05 was considered to be statistically significant. All values are expressed as the mean ± SEM.

## Results

### Exercise improved insulin sensitivity in the OLETF rats

We first confirmed that exercise improves insulin sensitivity in the OLETF rats under the experimental conditions. The OLETF and LETO rats were allowed to exercise voluntarily on the wheel placed in the cages for 20 weeks. Consequently, significant differences were found in the whole-body weight and epididymal fat weight of the OLEFT rats at 25 weeks of age after overnight fasting compared to those observed in the LETO rats under sedentary conditions (S1A to S1C Fig). The voluntary exercise significantly increased food intake normalized to body weight in both OLETF and LETO rats compared with sedentary condition (S1D Fig). This resulted in the total wheel running distance of 596.5 ± 39.1 km (mean + SEM) in the OLEFT rats and that of 709.1 ± 91.6 km in the LETO rats. Although the running distance appeared to be greater in the LETO rats than the OLEFT rats, there was no significant difference (S1E Fig).

**Fig 1:**
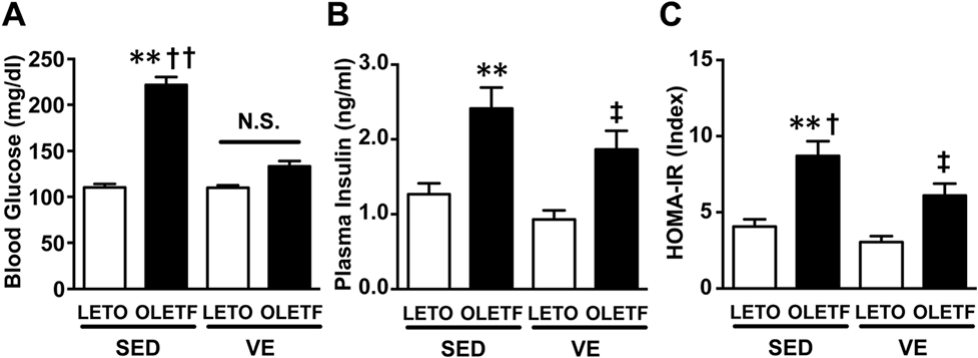
Hyperglycemia and hyperinsulinemia in the OLETF rats were reversed by voluntary exercise. The blood glucose (**A**) and plasma insulin (**B**) levels were significantly greater in the OLETF rats under sedentary conditions (SED) than in the LETO rats on SED. There were no significant differences between the LETO and OLETF rats with respect to the effects of voluntary exercise (VE) on the plasma insulin levels. The OLETF-SED rats exhibited insulin resistance, as indicated by elevated HOMA insulin resistance index values (**C**). All values are presented as the mean ± SEM. n = 9–11 per group, *,p<0.05; **,p<0.01 versus sedentary LETO, †,p<0.05 versus voluntary exercise OLETF, ‡,p<0.05 versus voluntary exercise LETO. N.S.: not significant.

The blood glucose levels were significantly greater in the OLETF rats than in the LETO rats. Exercise decreased the fasting blood glucose levels in the OLETF rats to the values noted in the normal control rats (Fig 1A). Exercise also tended to decrease the fasting plasma insulin levels in the OLETF rats, but there was no statistical significance (Fig 1B). The homeostasis model assessment insulin resistance (HOMA2-IR) index values revealed that the exercise improved insulin sensitivity in the OLETF rats by 25% (Fig 1C). Exercise did not change the fasting blood glucose or insulin levels in the LETO rats. In contrast, the intraperitoneal glucose tolerance tests (ipGTTs) confirmed that glucose tolerance was impaired in the OLETF rats and subsequently normalized by exercise (S2 Fig).

Among factors that potentially affect insulin sensitivity, the body weight values were significantly greater in the OLETF rats than in the LETO rats. In addition, exercise decreased the body weight values in both the OLETF and LETO rats. Meanwhile, the amount of epididymal fat was significantly greater in the OLETF rats than in the LETO rats, and exercise decreased to the similar weight of epididymal fat in the OLETF and LETO rats. Finally, exercise reduced the level of food intake in the OLETF rats and increased the level of food intake in the LETO rats. The running distance was not significantly different between the OLETF and LETO rats.

### Exercise prevented the expression of iNOS and subsequent S-nitrosylation of Akt in the OLETF rats

**Fig 2:**
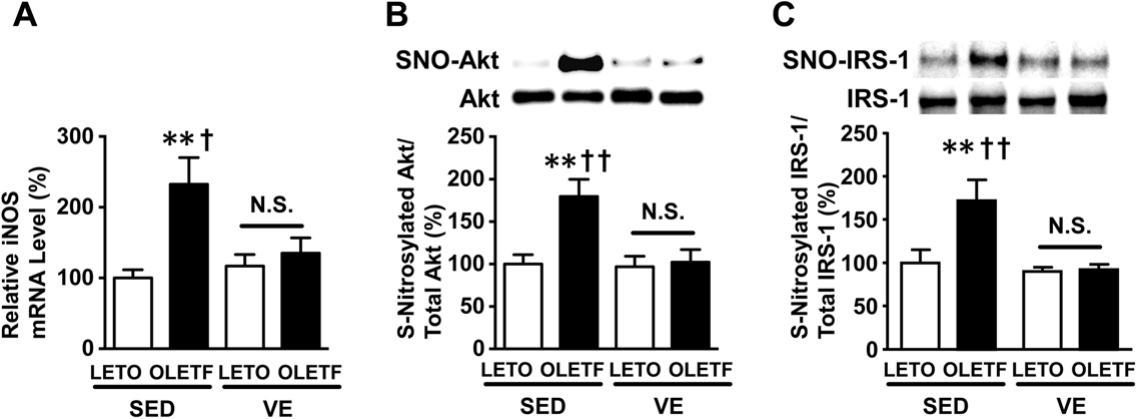
Effects of voluntary exercise on the iNOS mRNA expression and S-nitrosylation of Akt and IRS-1 in the liver in the OLETF rats. The mRNA expression of iNOS was significantly increased in the liver in the sedentary OLETF rats (**A**). The S-nitrosylated Akt levels were significantly increased in the liver in the sedentary (SED) OLETF rats (**B**). Voluntary exercise decreased the S-nitrosylated Akt levels in the liver in the voluntary exercise (VE) OLETF rats compared with those observed in the OLETF-SED rats. Similarly, S-nitrosylation of IRS-1 was also increased in the liver of SED OLETF rats (**C**). The degree of S-nitrosylation was evaluated using a biotin switch analysis. All values are presented as the mean ± SEM. n = 9–11 per group, **,p<0.01 versus sedentary LETO, †,p<0.05; ††,p<0.01 versus voluntary exercise OLETF. N.S.: not significant.

The expression of iNOS mRNA was significantly increased in the liver in the sedentary OLETF rats compared with that observed in the counterpart sedentary LETO rats (Fig 2A). The increased iNOS expression in the OLETF rats was significantly suppressed by voluntary exercise (Fig 2A).

The S-nitrosylation of Akt was markedly increased in the liver in the OLETF rats compared with that seen in the LETO rats (Fig 2B). In addition, exercise significantly decreased the degree of S-nitrosylated Akt in the liver in the OLETF rats, while it did not alter Akt protein abundance (Fig 2B). Similarly, S-nitrosylation of IRS-1 was also increased in liver of OLETF rats under sedentary condition (Fig 2C), consistent with previous studies in skeletal muscle of obese, diabetic mice [7, 30, 31]. Total IRS-1 expression was not altered by obesity or voluntary exercise (Fig 2C)

**Fig 3:**
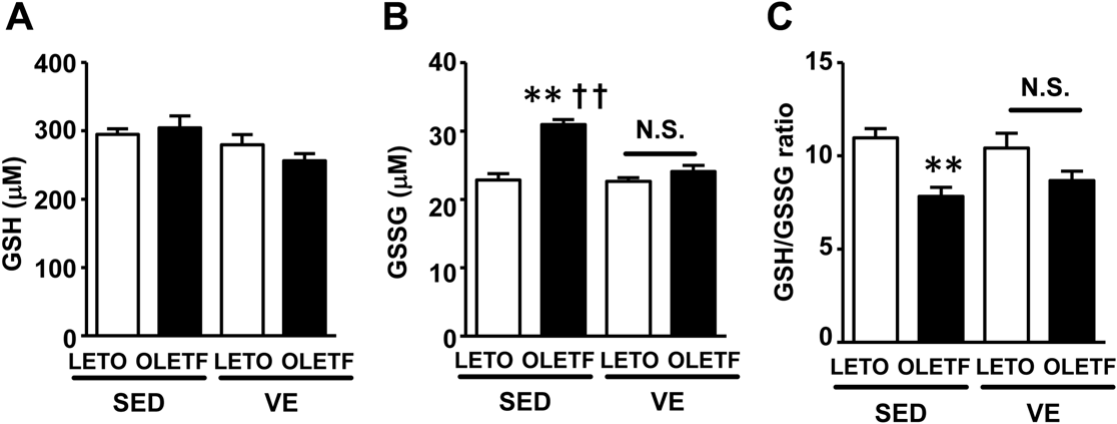
Voluntary exercise did not affect the GSH/GSSG ratio in the liver. Liver GSH content(**A**). The liver GSSG content was significantly greater in the OLETF-SED rats than in the LETO-SED rats (**B**). The GSH/GSSG ratio in the liver did not change with voluntary regular exercise in either the LETO or OLETF rats compared with their respective sedentary counterparts (**C**). All values are presented as the mean ± SEM. n = 9–11 per group, **,p<0.01 versus sedentary LETO, ††,p<0.01 versus voluntary exercise OLETF. N.S.: not significant.

GSH facilitates denitrosylation, and therefore an increase in the GSH level supposedly reduces the S-nitrosylation of Akt. However, contrary to our expectation, voluntary exercise did not increase the GSH content or GSH/GSSG ratio in the liver in either the LETO or OLETF rats (Fig 3A and 3C). Meanwhile, the GSSG content, which reflects oxidative stress, was significantly greater in the OLETF rats under sedentary conditions and was significantly decreased by voluntary exercise (Fig 3B). Concomitant oxidative stress enhances protein S-nitrosylation [32]. It is conceivable, therefore that iNOS induction and oxidative stress may contribute in concert to the increased Akt S-nitrosylation in the liver of OLEFT rats.

### Exercise reduced the liver triglyceride content and decreased the phosphorylation of c-jun N-terminal kinase (JNK) and insulin receptor substrate-1 (IRS-1) in the OLETF rats

In order to assess other mechanisms involved in the pathogenesis of hepatic insulin resistance, we evaluated the triglyceride content and the expression of molecules that participate in lipogenesis in the liver, such as sterol-regulatory element binding protein-1 (*Srebp-1*), stearoyl coenzyme A desaturase-1 (*Scd-1*), acetyl-CoA carboxylase (*Acc*), fatty acid synthase (*Fas*) and glycerol-3-phosphate acyltransferase 2 (*Gpat2*).

**Fig 4:**
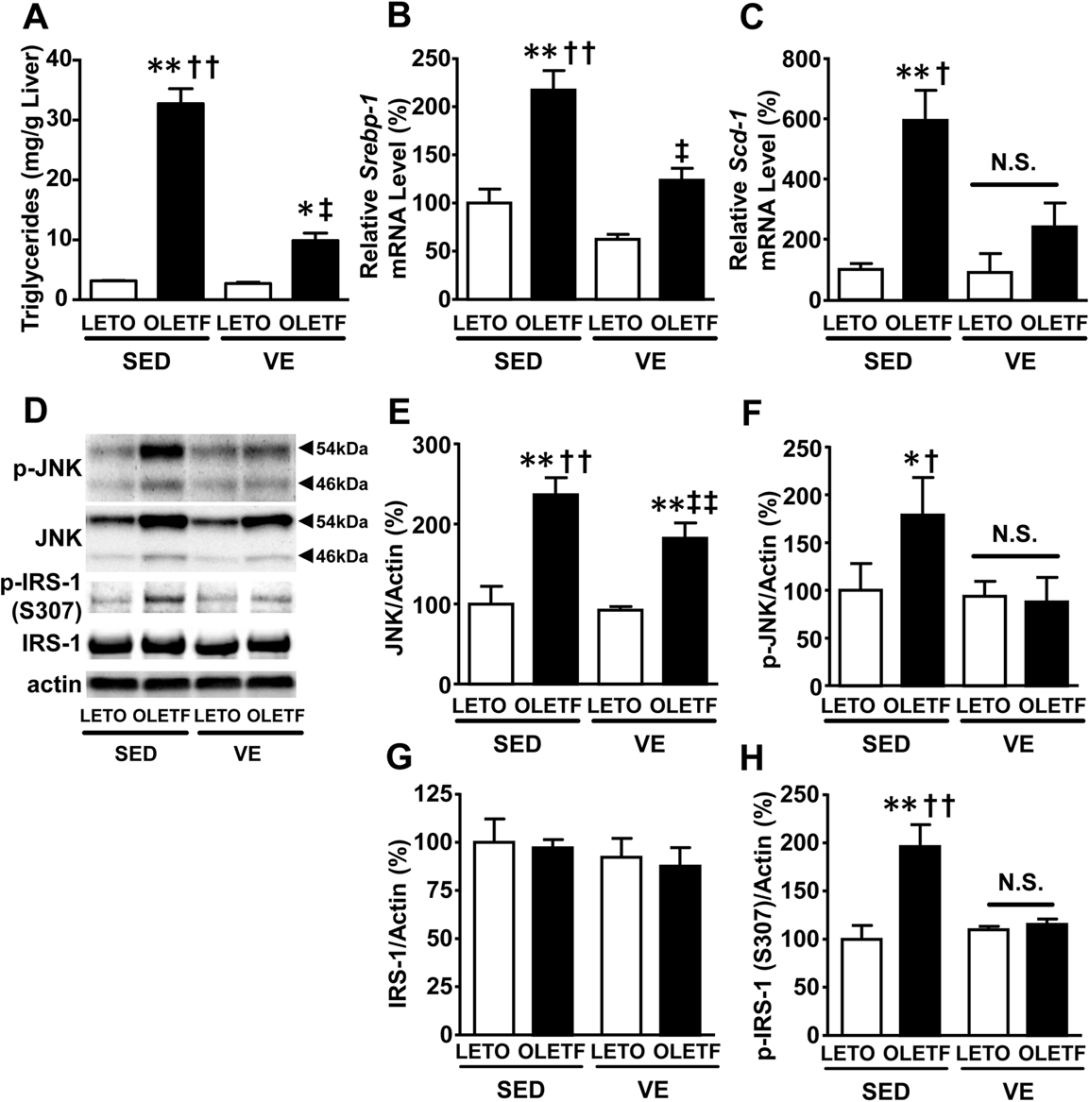
Exercise suppressed the lipogenic gene expression and prevented the accumulation of TG and activation of JNK in the liver in the OLETF rats. The triglyceride content in the liver (**A**) was significantly lower in the voluntary exercise (VE) OLETF rats than in the sedentary (SED) OLETF rats. Consistent with the decreased triglyceride levels, the mRNA expression of *Srebp-1* (**B**) and *Scd-1* (**C**) was significantly decreased in the OLETF-VE rats. It is known that lipid accumulation in the liver increases the JNK activity. The total JNK amount was significantly increased in the OLETF rats compare with that observed in the LETO rats (**D** and **E**). The phosphorylation of JNK was significantly increased in the liver in the OLETF-SED rats compared with that observed in the LETO-SED rats (**D** and **F**). After 20 weeks of exercise, the activated JNK content in the liver decreased in the OLETF rats. The protein levels of JNK and p-JNK were normalized to that of actin. All values are presented as the mean ± SEM. n = 7–11 per group, *,p<0.05; **,p<0.01 versus sedentary LETO, †,p<0.05; ††,p<0.01 versus voluntary exercise OLETF. ‡,p<0.05; ‡‡,p<0.01 versus voluntary exercise LETO. N.S.: not significant.

Notably, the triglyceride content was significantly greater in the liver in the OLETF rats than in the LETO rats (Fig 4A). Exercise decreased the TG content in the liver in the OLETF, but not LETO, rats (Fig 4A). Concordantly, the amount of mRNA for *Srebp-1* and *Scd-1* was greater in the liver in the OLETF rats than in the LETO rats, and exercise decrease the amount of this mRNA in the OLETF, but not LETO, rats (Fig 4B and 4C). In contrast, the amount of mRNA for *Acc*, *Fas* and *Gpat2*, which are partly regulated by *Srebp-1* and play important roles in hepatic steatosis, did not differ between the OLETF and LETO rats (S3 Fig). Exercise did not affect the amount of mRNA for these molecules in either the OLETF or LETO rats. Hepatic steatosis is associated with the activation of JNK, which mediates inflammatory signals [33, 34]. Western blot analysis revealed that the amount of both total and phosphorylated JNK was greater in the OLETF rats than in the LETO rats under sedentary conditions (Fig 4D and 4E). Exercise consequently suppressed the phosphorylation of JNK, while slightly decreasing the amount of total JNK proteins (Fig 4F).

Activation of the JNK pathway induces insulin resistance and JNK phosphorylates IRS-1 at serine 307 [35–37]. We, therefore, assessed phosphorylation status of IRS-1. Phosphorylation of IRS-1 at serine 307 was significantly increased in the liver of OLETF rats under sedentary condition, which paralleled increased phosphorylation (activation) of JNK. Voluntary exercise significantly decreased phosphorylation of serine 307 in IRS-1 as well as JNK phosphorylation (Fig 4F and 4H), while IRS-1 protein abundance was not altered (Fig 4G).

### Improved hepatic insulin signaling in OLETF rats after voluntary exercise

**Fig 5:**
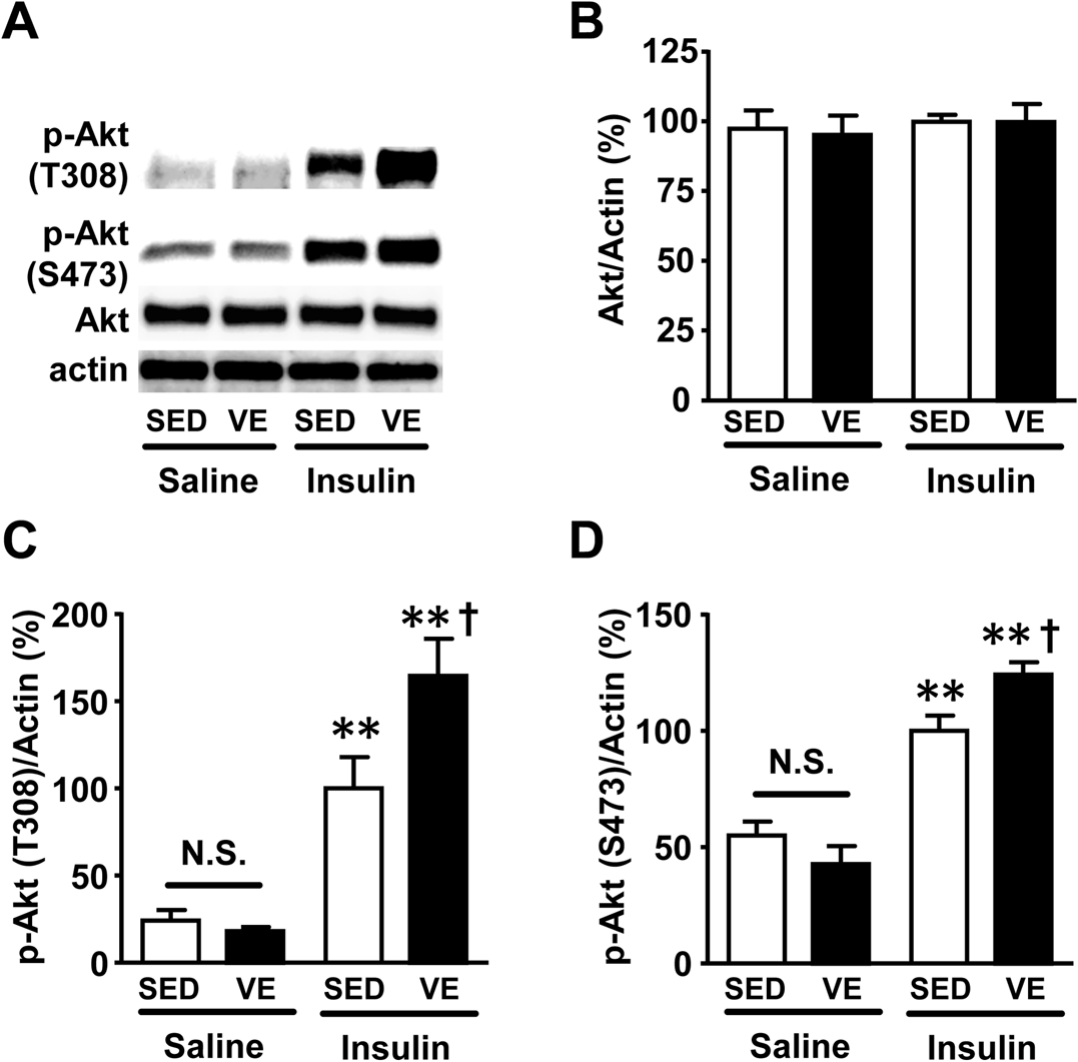
Insulin-stimulated phosphorylation of Akt was significantly improved in liver of OLETF rats after voluntary exercise. At 15 weeks of age, insulin (0.5 U/kg BW) or saline was injected via the portal vein following overnight fasting. At 5 min after the injection, liver was taken under anesthesia. Insulin-stimulated phosphorylation of Akt at threonine 308 (C) and serine 473 (D) was significantly increased in the liver of OLETF rats after exercise, as compared with sedentary condition. Basal (exogenous insulin-naïve) Akt phosphorylation in the liver was not different between the voluntary exercise and sedentary OLETF rat groups. The protein expression of Akt did not differ between voluntary exercise and sedentary condition in the liver of OLETF rats (B). *,p<0.05; **,p<0.01 versus sedentary OLETF with saline, †,p<0.05 versus sedentary OLETF with insulin. N.S.: not significant.

Next, we examined the effects of voluntary exercise on insulin signaling. Insulin-stimulated phosphorylation of Akt at threonine 308 and serine 473 was significantly increased in the liver of voluntary exercise OLETF rats compared to the sedentary group (Fig 5). The protein abundance of Akt did not differ between the voluntary exercise and sedentary groups (Fig 5B). Basal (exogenous insulin-naïve) Akt phosphorylation seems to be decreased by voluntary exercise in OLETF rats, but there was no statistically significant difference between the voluntary exercise and sedentary OLETF groups (Fig 5C and 5D). Body weight, blood glucose and plasma insulin levels after overnight fasting were significantly decreased in the voluntary exercise group compared with the sedentary group in OLEFT rats (S4A to S4C Fig).

## Discussion

In this report, we showed that voluntary exercise ameliorates insulin resistance, at least partly, by reducing the iNOS expression and the level of S-nitrosylated Akt in the liver. Voluntary exercise also decreases oxidative stress, hepatic steatosis and JNK activation, all of which contribute to suppressing chronic low-level inflammation in the liver and improve systemic insulin resistance.

To the best of our knowledge, this is the first report to show the mechanisms underlying improvements in hepatic insulin resistance induced by voluntary exercise. The favorable effects of exercise on glucose metabolism have been reported in rodents and humans, and, in most cases, these effects are attributed to reductions in insulin resistance in the skeletal muscle [38–40]. We previously showed that the forced expression of iNOS in the liver is sufficient to develop systemic insulin resistance and hyperglycemia in mice [9]. The excessive NO production by iNOS along with concomitant oxidative stress induces S-nitrosylation of Akt [8], a key player in the metabolic actions of insulin including insulin-stimulated glucose uptake [41]. We and others have previously reported that S-nitrosylation inactivates Akt [7, 32, 42], which in turn leads to insulin resistance in muscle and the liver. More specifically, in the liver-specific iNOS transgenic mice, increased Akt S-nitrosylation was associated with impaired insulin signaling and hyperglycemia [9]. In OLETF rats, the iNOS expression is enhanced and a key molecule, Akt, is S-nitrosylated, indicating that hepatic insulin resistance resulting from iNOS-induced S-nitrosylation plays a role in the onset of systemic insulin resistance in OLETF rats. Twenty weeks of voluntary exercise normalizes insulin resistance, the iNOS expression and the S-nitrosylation of Akt simultaneously, thus supporting the idea that voluntary exercise ameliorates insulin resistance, at least partly, by reducing the iNOS expression and the reversal of Akt S-nitrosylation in the liver.

In addition to Akt S-nitrosylation, S-nitrosylation of IRS-1 was increased in the sedentary OLEFT rats, similar to that in the liver-specific iNOS transgenic mice [9]. It is possible, therefore, that S-nitrosylation of IRS-1 may work in concert with S-nitrosylation of Akt to the insulin resistance in sedentary OLETF rats.

Moreover, our previous study has shown that increased iNOS expression is sufficient to cause increases in JNK phosphorylation (activity) and triglycerides content in the liver [9]. Together, there appears to be a vicious cycle involving S-nitrosylation and other mechanisms of insulin resistance, such as hepatic steatosis and activation of JNK. Fat accumulation in the liver causes insulin resistance [43, 44] and induces inflammation in the liver, which in turn stimulates the expression iNOS and increases S-nitrosylation [6, 45, 46]. On the other hand, iNOS induction and subsequent insulin resistance result in fat accumulation in the liver by activating the JNK pathway [9, 34, 47–49]. The activation of JNK in the liver enhances inflammation, iNOS production and hepatic insulin resistance [50, 51], while the expression of iNOS induces JNK activation in the liver [9]. In fact, in the present study, exercise decreased S-nitrosylation as well as the levels of both TG and activated JNK. JNK activation plays an important role in the development of obesity-induced insulin resistance [50]. In addition, previous studies have reported that phosphorylation of IRS-1 at serine 307, a JNK phosphorylation site, is increased in obesity-induced insulin resistance [35–37, 52]. Similarly, we found that phosphorylation of serine 307 in IRS-1 was increased in sedentary OLETF rat relative to LETO rats. Importantly, voluntary exercise reduced phosphorylation of IRS-1 at serine 307 in OLETF rats to the levels observed in LETO rats (Fig 4H). From a mechanistic point of view, however, controversial results have been reported about whether phosphorylation of serine 307 in IRS-1 mediates insulin resistance [52, 53]. Regardless, our data suggest that iNOS-involved JNK activation in sedentary OLEFT rats and its amelioration by voluntary exercise may play a role in the insulin resistance and its improvement.

Our previous study showed that the expression of iNOS in the liver is sufficient to induce systemic insulin resistance [9], while the inhibition of iNOS blocks this vicious cycle and improves insulin resistance [8, 27]. In OLETF rats, voluntary exercise significantly improved insulin-stimulated Akt phosphorylation compared to sedentary OLETF rats. These effects of voluntary exercise are associated with suppressed inflammatory response in the liver, such as decreased iNOS mRNA levels. These results are consistent with our previous reports [8, 9]. Our findings, together with the previous studies conducted by our group and others, strongly suggest that iNOS plays an important role in exercise-induced improvements in insulin resistance.

The relative importance of S-nitrosylation of act in the liver and other proposed mechanisms underlying the exercise-induced improvement of systemic insulin resistance remain to be elucidated. Exercise improves insulin resistance in the skeletal muscle via various mechanisms, including the mechanical stretch-induced activation of AMP-activated kinase [54], changes in energy metabolism [55], decreases in the iNOS expression and S-nitrosylation [56, 57], and reductions in the fat content in the muscle [58]. Exercise also decreases the level of food intake and suppresses obesity in OLETF rats [59, 60]. Moreover, exercise suppresses inflammation in the liver as well as other parts of the body in OLETF rats [61–64]. It is therefore likely that the exercise-induced changes in S-nitrosylation and the iNOS expression observed in the liver contribute to improve insulin resistance in addition to these other mechanisms.

In conclusion, voluntary exercise induces a cascade of events, including the decreases in the triglyceride content, the iNOS expression, the S-nitrosylation of Akt and IRS-1, and the phosphorylation (activation) of JNK, leading to the improved insulin sensitivity in the liver of OLETF rats.

## Supporting Information

S1 Fig Regular exercise prevented obesity in the OLETF rats. Five-week old male LETO and OLETF rats were randomly assigned to a sedentary (SED) or voluntary exercise (VE) group and their body weights and food intake were recorded for 20 weeks (**A**). Starting at 8 weeks of age, the body weights of the OLETF-SED rats were significantly greater than those of the LETO-SED rats. *,p<0.05 ORETF-SED versus LETO-SED. (**B**, **C**) The body weight (**B**) and epididymal fat weight on both sides (**C**) were significantly greater in OLEFT-SED rats than LETO rats at 25 weeks of age, which were reversed by voluntary exercise. (**D**) Average food intake normalized to body weight did not significantly differ between LETO and OLETF rats. Voluntary exercise significantly increased food intake normalized to body weight in both LETO and OLETF rats relative to the respective sedentary counterparts. (**E**) Average daily wheel running distance was not significantly different between the LETO-VE and OLETF-VE rats. All values are presented as the mean ± SEM. n = 9–11 per group, *,p<0.05; **,p<0.01 versus sedentary LETO, †,p<0.05 versus voluntary exercise OLETF, ‡,p<0.05 versus voluntary exercise LETO. N.S.: not significant.
(TIF)

S2 Fig ipGTT revealed that exercise prevents insulin resistance. Glucose tolerance test (GTT, 1.0 g/kg body weight, intraperitoneal injection) demonstrated glucose intolerance in the OLETF rats compared with the LETO rats (**A**). The area under the glucose curve was calculated during GTT (**B**). All values are presented as the mean ± SEM. n = 9–11 per group, *,p<0.05; **,p<0.01 versus sedentary LETO, †,p<0.05 versus voluntary exercise OLETF. N.S.: not significant.
(TIF)

S3 Fig Lipogenic gene mRNA expression in the liver. The mRNA expression levels of acetyl-CoA carboxylase (*Acc*; **A**), fatty acid synthase (*Fas*; **B**) and glycerol-3-phosphate acyltransferase 2 (*Gpat2*; **C**) were not significantly different between thew groups. All values are presented as the mean ± SEM. n = 9–11 per group.
(TIF)

S4 Fig Body weight, blood glucose and plasma insulin were significantly decreased in OLETF rats after voluntary exercise. At five weeks of age, the OLETF rats were randomly assigned to a sedentary or voluntary exercise group. After 10-week voluntary exercise, body weight (A), blood glucose (B) and plasma insulin (C) were significantly decreased in voluntary exercise group (VE) compared with sedentary group (SED). All values are presented as the mean ± SEM. n = 5–7 per group, **,p<0.01 versus sedentary group.
(TIF)
