# Supplementary figures and images for: Voluntary Exercise Can Ameliorate Insulin Resistance by Reducing iNOS-Mediated S-Nitrosylation of Akt in the Liver in Obese Rats
Source: PLoS One. 2015 Jul 14;10(7):e0132029. doi: 10.1371/journal.pone.0132029 (PMC4501761; doi:10.1371/journal.pone.0132029)

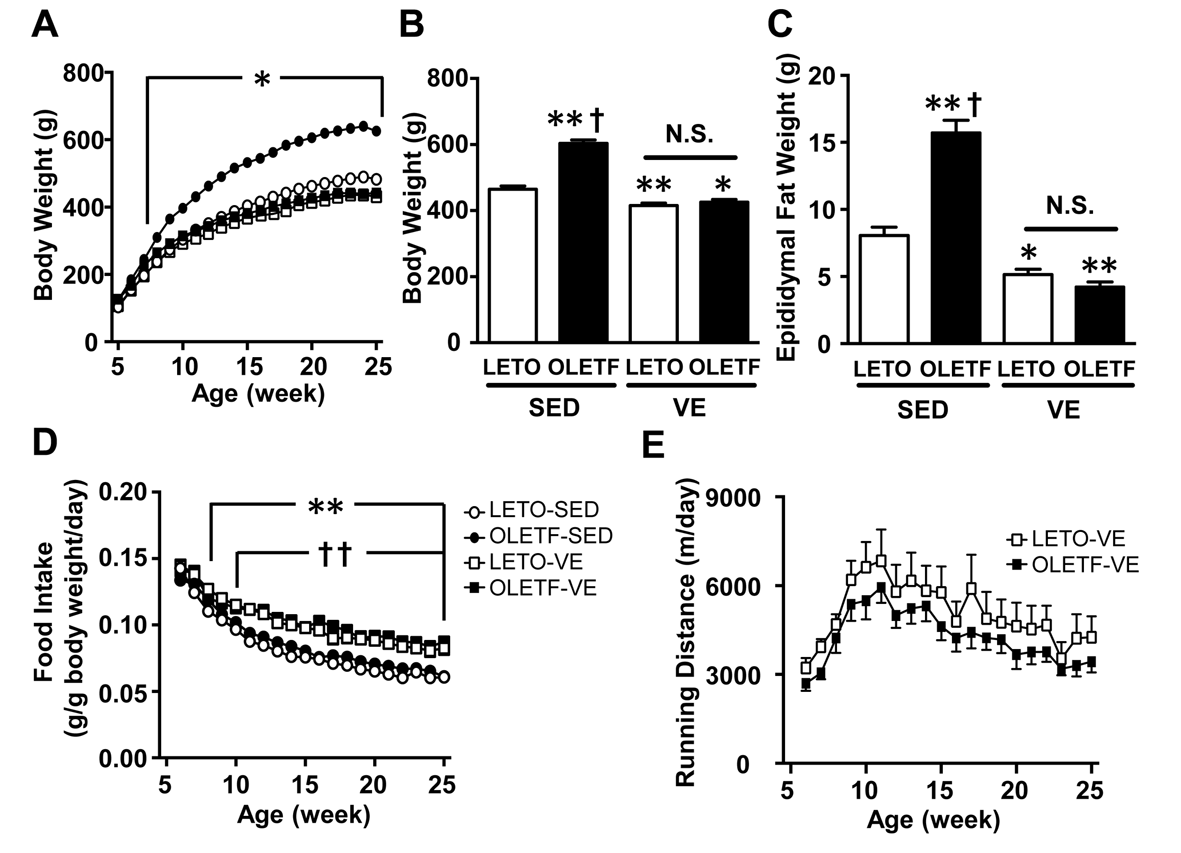

Supplement: S1 Fig — Five-week old male LETO and OLETF rats were randomly assigned to a sedentary (SED) or voluntary exercise (VE) group and their body weights and food intake were recorded for 20 weeks (A). Starting at 8 weeks of age, the body weights of the OLETF-SED rats were significantly greater than those of the LETO-SED rats. *,p<0.05 ORETF-SED versus LETO-SED. (B, C) The body weight (B) and epididymal fat weight on both sides (C) were significantly greater in OLEFT-SED rats than LETO rats at 25 weeks of age, which were reversed by voluntary exercise. (D) Average food intake normalized to body weight did not significantly differ between LETO and OLETF rats. Voluntary exercise significantly increased food intake normalized to body weight in both LETO and OLETF rats relative to the respective sedentary counterparts. (E) Average daily wheel running distance was not significantly different between the LETO-VE and OLETF-VE rats. All values are presented as the mean ± SEM. n = 9–11 per group, *,p<0.05; **,p<0.01 versus sedentary LETO, †,p<0.05 versus voluntary exercise OLETF, ‡,p<0.05 versus voluntary exercise LETO. N.S.: not significant. (TIF) [file pone.0132029.s001.tif]

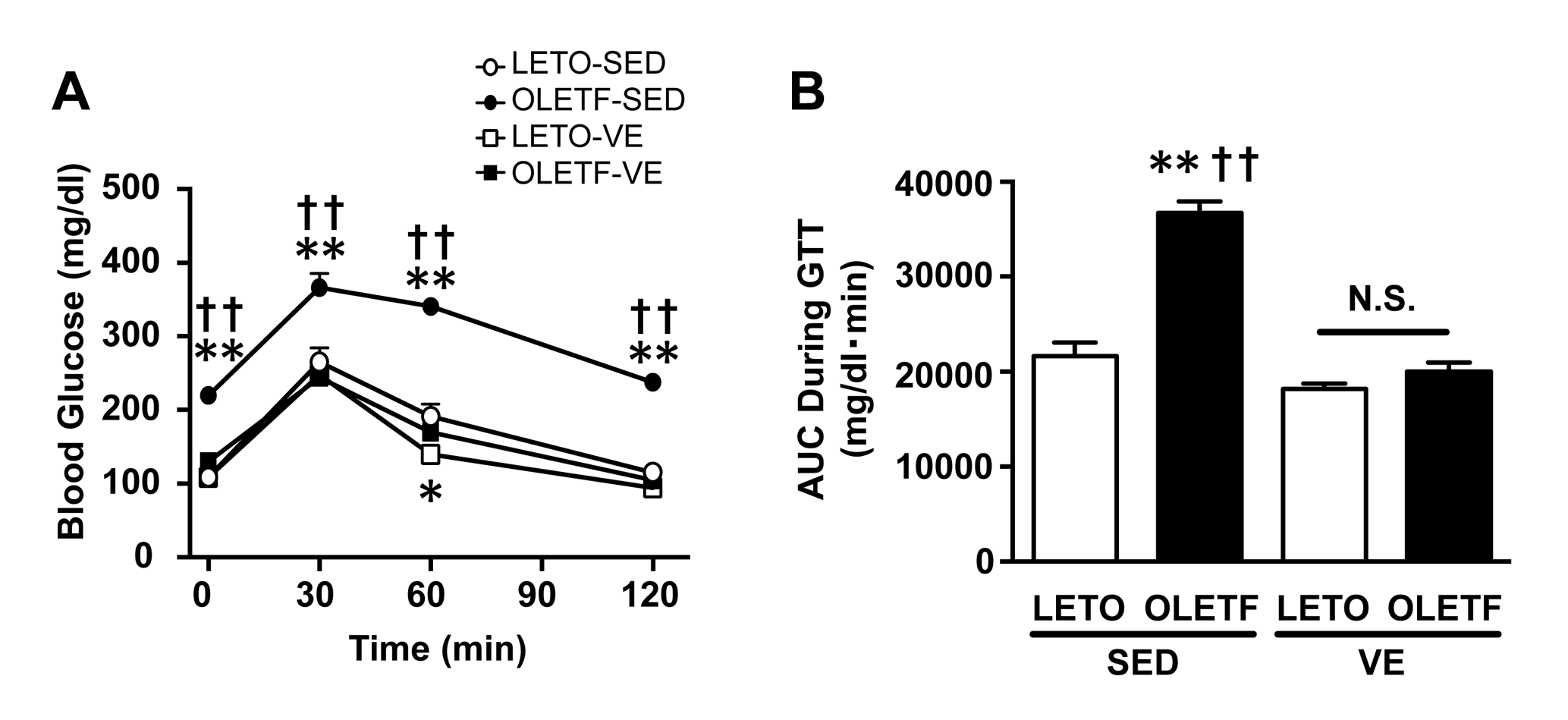

Supplement: S2 Fig — Glucose tolerance test (GTT, 1.0 g/kg body weight, intraperitoneal injection) demonstrated glucose intolerance in the OLETF rats compared with the LETO rats (A). The area under the glucose curve was calculated during GTT (B). All values are presented as the mean ± SEM. n = 9–11 per group, *,p<0.05; **,p<0.01 versus sedentary LETO, †,p<0.05 versus voluntary exercise OLETF. N.S.: not significant. (TIF) [file pone.0132029.s002.tif]

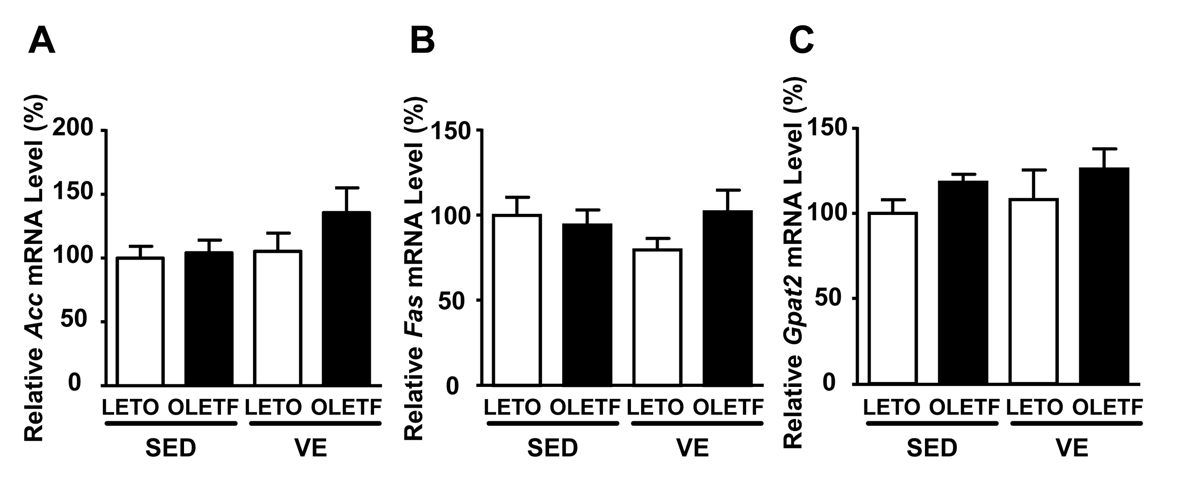

Supplement: S3 Fig — The mRNA expression levels of acetyl-CoA carboxylase (Acc; A), fatty acid synthase (Fas; B) and glycerol-3-phosphate acyltransferase 2 (Gpat2; C) were not significantly different between thew groups. All values are presented as the mean ± SEM. n = 9–11 per group. (TIF) [file pone.0132029.s003.tif]

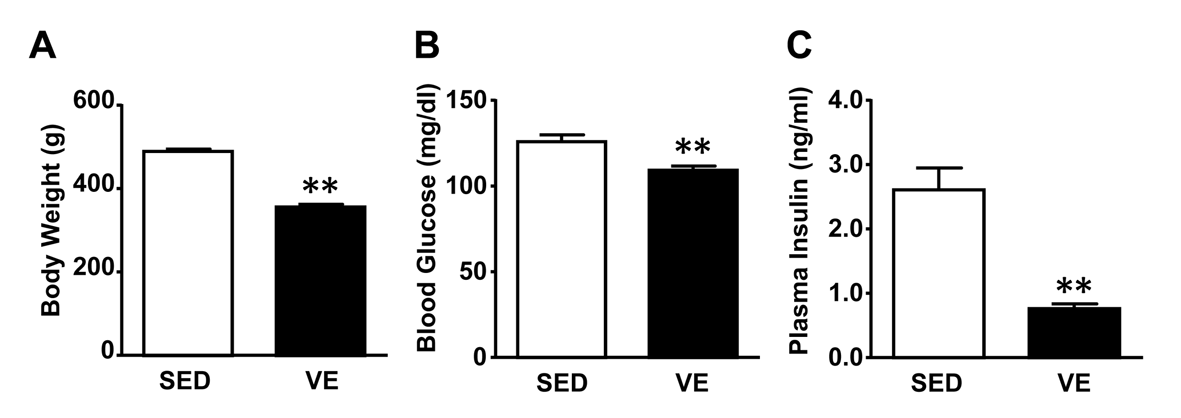

Supplement: S4 Fig — At five weeks of age, the OLETF rats were randomly assigned to a sedentary or voluntary exercise group. After 10-week voluntary exercise, body weight (A), blood glucose (B) and plasma insulin (C) were significantly decreased in voluntary exercise group (VE) compared with sedentary group (SED). All values are presented as the mean ± SEM. n = 5–7 per group, **,p<0.01 versus sedentary group. (TIF) [file pone.0132029.s004.tif]
